# Theory Design of a Virtual Polarizer with Multiscale and Multi-Biomass Sensing

**DOI:** 10.3390/bios15080516

**Published:** 2025-08-08

**Authors:** Chuanqi Wu, Haifeng Zhang

**Affiliations:** College of Electronic and Optical Engineering & College of Flexible Electronics (Future Technology), Nanjing University of Posts and Telecommunications, Nanjing 210023, China; b22021517@njupt.edu.cn

**Keywords:** virtual polarizer, multi-biomass sensing, multiscale sensing, coherent perfect polarization control, biosensing

## Abstract

Recently, more and more attention has been paid to human health with the rapid development of society. A designed virtual polarizer (VP) can realize multiscale and multi-biomass sensing, including temperature, cancerous cells, and COVID-19. Based on coherent perfect polarization conversion, a certain polarization conversion can be fulfilled within 1.72~2.14 THz. Then, through observing the displacement of a perfect matching point (PMP), variations in temperature can be accurately determined, covering from 299 K to 315 K, with a sensitivity (*S*) of 0.0198 THz/K. Moreover, a sharp coherent perfect absorption (CPA) peak generated from the VP can be employed for the detection of cancerous cells and COVID-19. The refractive index (RI) detection range of cancerous cells is from 1.36 RIU to 1.41 RIU with the sensitivity being −4.45881 THz/RIU. The average quality factor (*Q*), figure of merit (*FOM*), and detection limit (*DL*) are 825.36, 241.11 RIU^−1^, and −36.83 dB. For the COVID-19 solution concentration (SC) from 0 mM to 525 mM, by mapping SC to RI, the RI sensing range is 1.344 RIU–1.355 RIU with the *S* being −5.03467 THz/RIU. The relevant *Q*, *FOM*, and *DL* are 760.85, 244.94 RIU^−1^, and −36.89 dB. Based on two strategies of PMP and CPA, the proposed VP is capable of multiscale and multi-biomass sensing with excellent detection performance, providing a new detection method for biosensing.

## 1. Introduction

The phenomenon of interference, employed for several devices like spectrometers and antennas, will occur when several coherent waves overlap with each other, causing the redistribution of energy in space [1]. Based on the interference principle of electromagnetic (EM) waves, resonators can realize the complete absorption of incident EM waves when critical coupling is realized by adjusting the loss rate in the device, which is usually thought of as coherent perfect absorption (CPA) [2]. Based on the time-reversal process of laser emission [3,4], CPA is usually evaluated by the scattering matrix, where CPA can take place only when the characteristic values of the scattering matrix are equal to zero [5]. As a result, most situations of CPA will occur in a narrow frequency band, making CPA have great potential in detecting tiny changes in external physical quantities [6]. Moreover, with a similar efficiency and coherent nature to CPA, coherent perfect polarization control (CPPC) represents a conservative and reversible optical mode conversion process, which is often realized in a multi-port system [6]. The polarization state of EM waves can be precisely adjusted by CPPC within a certain frequency range [7]. Devices with the functions of CPA and CPPC can be described as virtual polarizers (VPs) [6,7]. The potential of CPA and CPPC in the multiscale and multi-biomass sensing field is the important in this paper.

Nowadays, cancer, as one of the most widely distributed diseases, has more than 200 different types and affects over than 60 human organs [8], which always greatly damages human health and must be prevented early. Given that the research about EM waves is developing rapidly, EM devices have great potential in polarization control, optic encoding, beam control, etc. [9,10,11,12,13], where more and more attention is paid to the exploration of biosensing based on the nature of EM waves. Due to cancerous cells usually having a higher protein concentration than normal cells, the refractive index (RI) of a normal cell is smaller than that of a cancerous cell [14], which serves as an important indicator for early cancer cell detection. In 2022, Alizadeh et al. proposed a graphene-based RI sensor, which can utilize an absorption displacement peak to detect different parts of body fluids and distinguish normal and cancerous cells [15]. The corresponding sensitivity (*S*) and quality factor (*Q*) of cancerous cells are 3 THz/RIU and 15. In 2025, Banerjee et al. designed a THz metamaterial absorber to sense skin cancer and used machine learning to improve the sensing performance [16], where the *S* and *Q* are equal to 0.3 THz/RIU and 61.75. Apart from the threat of cancer to human health, against the background of deep globalization, there are more and more chances to make unknown and new viruses have the ability to spread rapidly all over the world [17], resulting in a great threat to public health and the global economy. Therefore, it is urgent to propose a direct-testing, fast-response, and universally applicable method to detect viruses. In 2022, Ghosh et al. designed a broadband biosensor using a graphene-metasurface-based cross-polarization converter, the core principle of which is to build a certain relationship between the changes in polarization conversion rate and the variations in virus concentrations [18]. In 2023, Upender et al. presented a tunable dual-band THz metamaterial sensor to detect six different types of viruses with the *S* being 3.29 THz/RIU and 3.28 THz/RIU [19], the key principle of which is to utilize two absorption displacement peak located in two different frequency band to realize the detection of viruses. The related Q are 480 and 371.42, respectively. Although the above studies can effectively detect certain cancerous cells or viruses with excellent sensing performance, the detection strategy of these studies tends to be single and can not form a multiscale sensing function. Moreover, most studies of sensing cancerous cells or viruses only focus on the detection of single biomass. Hence, it is necessary to combine multiple sensing strategies and increase the types of detected biomass to form multiscale and multi-biomass sensing.

In this paper, a VP with multiscale and multi-biomass sensing is proposed, which is composed of indium antimonide (InSb) [20], a graphene layer (GL) [21], detected layers, and two common media [22,23]. When two beams of coherent linear polarization waves (LPWs), respectively, incident from the antecedent and consequent of VP at the same time, the proposed VP will generate CPA or CPPC. When CPPC is generated, a certain polarization conversion from LPWs to right-handed circular polarization waves (RHCPWs) occurs within 1.72~2.14 THz. Here, the frequency point, corresponding to an axis ratio (*AR*) of 0 dB [6,7], is defined as a perfect matching point (PMP), meaning the output EM waves at this time are standard circular polarization waves (CPWs). Considering the temperature tunability of InAs, the PMP will linearly shift with temperature (*T_e_*) changing from 299 K to 315 K. When the state of the given VP is switched from CPPC to CPA, a narrow CPA peak will take place under the TM mode in the range of 15 THz~16 THz. Through injecting the detected object into the detected layers, the CPA peak will vary as the RI of the detected object changes, where a linear relationship between the CPA peak and the RI of the detected object is created. Here, the detected object can be six different cancerous cells or COVID-19 solution concentrations (SCs), the related ranges of which are, respectively, 1.36 RIU~1.41 RIU and 0 mM~525 mM. Notably, the variations in COVID-19 SC are mapped into the changes in RI for convenient sensing. Based on two sensing strategies of CPA and CPPC, the proposed VP has the ability of multiscale and multi-biomass sensing, which not only provides a distinctly ideal approach of enlarging the sensing scale but also has great potential in the field of biosensing.

## 2. Materials and Methods

**Figure 1:**
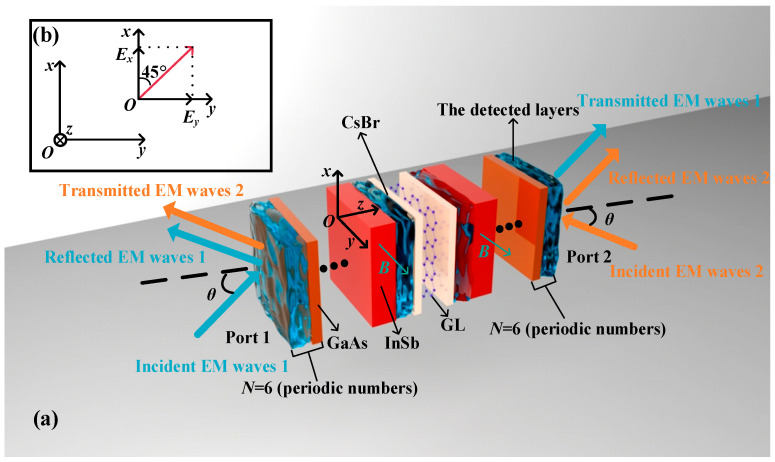
(**a**) The diagrams of the overall structure of the designed VP, where two beams of coherent EM waves incident from the preceding and the subsequent items of the VP. (**b**) The electric fields (*E^f^*) of EM waves 1 and 2 are at the angle of 45° to the +*x*-axis, which can be divided into two components: *E^f^_x_* and *E^f^_y_*.

The multiscale and multi-biomass sensing of the VP is based on two detection strategies, CPA and CPPC, the essence of which is the interference of coherent beams. First, ports 1 and 2 are defined as the forward and backward of the VP, respectively. As shown in Figure 1a, two beams of coherent waves incident from the forward and backward of the VP simultaneously, where there is a phase difference Δ*Φ* between incident EM waves 1 and 2. For the incident EM waves, the direction of their electric fields *E^f^* is all at an angle of 45°, which can be vectorized into two components *E^f^_x_* and *E^f^_y_* along the +*x*-axis and the +*y*-axis [20]. The specific situation is demonstrated in Figure 1b. Therefore, the incident coherence beams can be considered the vector synthesis of transverse electric (TE) and transverse magnetic (TM) waves [20,21], where the propagation natures of coherent waves can be divided into those of TE and TM waves. In the given model, the direction of electric fields is parallel to the +*y*-axis for TE waves [21]. Also, the direction of magnetic fields is parallel to the +*y*-axis for TM waves [21]. The phase difference between TE and TM waves is assumed as Δ*φ* = *φ*_TM_ − *φ*_TE_ [20].

The transmission characteristics of two beams of coherent waves can be calculated by the transfer matrix method of layered structure theory [20], which requires determining the structure parameters of the VP. Firstly, the VP consists of InSb [20], GL [22], gallium arsenide (GaAs) [23], cesium bromide (CsBr) [24], and cavities, defined as A, C, D, H, and E. The use of cavities is to act as the detected layers to hold detected objects. The total configuration of the VP can be expressed as {(ED)^6^AHECEHA(DE)^6^}, the related thickness of which can be represented by *d*_A_ = 8.5 μm, *d*_C_ = 0.34 nm, *d*_D_ = 1.9 μm, *d*_H_ = 1.55 μm, and *d*_E_ = 8.13 μm. Notably, the relative magnetic permeabilities of all media in the VP are all equal to 1. The RIs of GaAs and CsBr are determined as *n*_GaAs_ = 3.43 [23] and *n*_CsBr_ = 1.15 [24]. Especially, within 1.72–2.14 and 15–16 THz, the dispersion effects of GaAs and CsBr are very weak and can be ignored [23,24], which can be regarded as ideal materials. The GL is located in the center of the VP, and the dielectric constant is connected with the electrical conductivity *σ* [22]. Considering that the dielectric functions of InSb are influenced by the temperature *T_e_* and external magnetic fields *B* [25], the initial values of the *T_e_* and *B* are fixed as 300 K and 0.98 T. In addition, the incident angle *Θ* of EM waves is set as 0°.

When the external magnetic field, parallel to the +*y*-axis, is applied in the VP, the InSb will exhibit anisotropy, the dielectric functions of which can be written as follows [26,27]:

(1) $${\overset{⌢}{\varepsilon }}_{\mathrm{InSb}}=\left[\begin{array}{ccc} \varepsilon_{\mathrm{xx}} & 0 & {i\varepsilon }_{\mathrm{xz}}\\ 0 & \varepsilon_{\mathrm{yy}} & 0\\ -{i\varepsilon }_{\mathrm{xz}} & 0 & \varepsilon_{\mathrm{xx}} \end{array}\right],$$

(2) $$\varepsilon_{\mathrm{xx}}=\varepsilon_{\infty }(1-\frac{\omega_{p}^{2}(\omega +i\upsilon )}{\omega [{\left(\omega +i\upsilon \right)}^{2}-\omega_{c}^{2}]}),$$

(3) $$\varepsilon_{\mathrm{yy}}=\varepsilon_{\infty }(1-\frac{\omega_{p}^{2}}{\omega (\omega +i\upsilon )}),$$

(4) $$\varepsilon_{\mathrm{xz}}=\varepsilon_{\infty }\frac{-\omega_{p}^{2}\omega_{c}}{\omega [{\left(\omega +i\upsilon \right)}^{2}-\omega_{c}^{2}]}.$$

where *ω* = 2πf and *f* stands for the frequency of coherent waves. Moreover, *i* is defined as the imaginary unit. *ω_p_* = [*Ne*^2^/*ε*_0_ε_∞_m*]^1/2^ and *ω_c_ = eB*/m* are respectively defined as the plasma frequency and cyclotron frequency [20], where *e* and *ε*_0_ are the unit charge and the vacuum dielectric constant. In addition, m* = 0.015*m*_e_ and *υ* = 0.000001*ω_p_* with *m*_e_ and *υ* being the electronic quality and plasma collision frequency [28]. The *N* related to *ω_p_* can be obtained from Equation (5). The high-frequency limit permittivity can be determined as *ε_∞_* = 15.68 [20].

(5) $$N=5.76\times 10^{20}T_{e}^{1.5}\exp[-0.26/(2\times 8.625\times 10^{-5}\times T_{e})].$$

Since the dielectric constant of GL is influenced by the electrical conductivity, the specific forms of *ε*_GL_ and σ can be deemed as follows [22,29]:

(6) $$\sigma =\frac{{ie}^{2}k_{B}T_{e}}{\pi \hbar^{2}\left(\omega +i/\tau \right)}\left(\frac{\mu_{C}}{k_{B}T_{e}}+2\ln\left(e^{-\frac{\mu_{C}}{k_{B}T_{e}}}+1\right)\right)+\frac{{ie}^{2}}{4\pi \hbar }1n\left(\frac{2|\mu_{C}|-\hbar (\omega +i/\tau )}{2|\mu_{C}|-\hbar (\omega +i/\tau )}\right),$$

(7) $$\varepsilon_{\mathrm{GL}}=1+\frac{i\sigma }{{\omega \varepsilon }_{0}d_{\mathrm{GL}}}.$$

where *k*_B_ and *ℏ* are described as the Boltzmann constant and the reduced Planck constant. The relaxation time *τ* is fixed as 0.1 ps [22]. Moreover, the chemical potential *μ*_C_ is equal to 0.8 eV. It is emphasized that there are no adjacent elements to affect the electronic band structure of GL.

Notably, owing to the fact that the operating frequencies of the VP are 1.72–2.14 THz and 15–16 THz, the EM response of the GL in this paper is based on the surface conductivity model, where the Equation (6) can be derived from the well-known Kubo formula [22,29]. Moreover, graphene is regarded as an infinitesimally thin, local two-sided surface [22,29], with the diameter of a carbon atom being 0.34 nm. Considering that the GL of the VP is a single-layer graphene, its thickness *d*_GL_ is usually determined as 0.34 nm (equal to the diameter of a carbon atom), employed in many research [22,29].

For the GL, GaAs, CsBr, and detected layers under the TE and TM modes, their form of the transfer matrix is the same as that of InSb under the TE mode, which can be expressed as follows [30]:

(8) $$\begin{array}{l} M_{g}=\left[\begin{array}{cc} \cos(k_{gz}d_{g}) & -\frac{i}{\eta_{g}}\sin(k_{gz}d_{g})\\ -{i\eta }_{g}\sin(k_{gz}d_{g}) & \cos(k_{gz}d_{g}) \end{array}\right]\\ (g=\mathrm{InSb},\;\mathrm{GL},\;\mathrm{GaAs},\;\mathrm{CsBr},\;\mathrm{Cavity}). \end{array}$$

where *k_gz_* and *η_g_* are on behalf of the wave vector and admittance, which can be obtained from Ref. [30]

When the coherent waves are under the TM mode, the transfer matrix of InSb can be determined as follows [20,30]:

(9) $$M_{\mathrm{InSb}}=\left[\begin{array}{cc} \cos(k_{z}d_{\mathrm{InSb}})+\frac{k_{x}\varepsilon_{\mathrm{xz}}}{k_{z}\varepsilon_{xx}}\sin(k_{z}d_{\mathrm{InSb}}) & -\frac{i}{\eta_{\mathrm{InSb}}}[1+{\left(\frac{k_{x}\varepsilon_{xz}}{k_{z}\varepsilon_{xx}}\right)}^{2}]\sin(k_{z}d_{\mathrm{InSb}})\\ -{i\eta }_{\mathrm{InSb}}\sin(k_{z}d_{\mathrm{InSb}}) & \cos(k_{z}d_{\mathrm{InSb}})-\frac{k_{x}\varepsilon_{xz}}{k_{z}\varepsilon_{xx}}\sin(k_{z}d_{\mathrm{InSb}}) \end{array}\right].$$

where *k_x_*, *k_z_*, and *η*_InSb_ can be calculated according to Ref. [20].

Based on the derivation of the transfer matrices related to all media in the VP, the energy process of EM waves can be attained, where the total transfer matrix of the whole VP can be given as follows [30]:

(10) $$\begin{array}{ll} M_{\mathrm{total}} & ={\left(M_{E}M_{C}\right)}^{6}M_{A}M_{E}M_{D}M_{\mathrm{GL}}M_{D}M_{E}M_{A}{\left(M_{C}M_{E}\right)}^{6}\\ & =\left[\begin{array}{cc} M_{11} & M_{12}\\ M_{21} & M_{22} \end{array}\right]. \end{array}$$

The reflection *r* and transmission *t* coefficients can be described as follows [30]:

(11) $$r=\frac{\left(M_{11}+\eta_{0}M_{12})\eta_{0}-(M_{21}+\eta_{0}M_{22}\right)}{\left(M_{11}+\eta_{0}M_{12})\eta_{0}+(M_{21}+\eta_{0}M_{22}\right)},$$

(12) $$t=\frac{2\eta_{0}}{\left(M_{11}+\eta_{0}M_{12})\eta_{0}+(M_{21}+\eta_{0}M_{22}\right)}.$$

where *η*_0_ is defined as the vacuum admittance. The reflectance and transmittance can be derived as *R* = |*r*|^2^ and *T* = |*t*|^2^.

With the Δ*Φ* being 180°, two beams of coherent waves can be defined as *I*^+^ and *I*^−^ according to the propagation direction, where EM waves propagating along the +*z*-axis are *I*^+^ and those propagating along the −*z*-axis are *I*^−^. Similarly, the output EM waves along the +*z*-axis or the −*z*-axis are assumed as *Q*^+^ or *Q*^−^, respectively. The specific relationship between *I*^+^, *I*^−^, *Q*^+^, and *Q*^−^ can be written as follows [7]:

(13) $$\left(\begin{array}{c} Q^{+}\\ Q^{-} \end{array}\right)=S\left(\begin{array}{c} I^{+}\\ I^{-} \end{array}\right)=\left(\begin{array}{cc} t & r\\ r & t \end{array}\right)\left(\begin{array}{c} I^{+}\\ I^{-} \end{array}\right).$$

The relationship between incident and scattering EM waves can be further expressed as follows [7]:

(14) $$Q^{+}=t|I^{+}|+r|I^{-}|e^{i\Delta \Phi },$$

(15) $$Q^{-}=r|I^{+}|+t|I^{-}|e^{i\Delta \Phi }.$$

According to Equations (14) and (15), the absorptivity *A_c_* of two beams of coherent waves can be written as follows [6]:

(16) $$A_{c}=1-|Q^{+}{|}^{2}-|Q^{-}{|}^{2}.$$

Assuming the Δ*φ*1 as Δ*φ*1 = Arg(*t*)-Arg(*r*), the *A_c_* can be further deemed as follows [6]:

(17) $$A_{c}=1-{\left(|t|-|r|\right)}^{2}-2|t||r|\left(1+\frac{2|I^{+}||I^{-}|\cos(\Delta \varphi 1)\cos(\Delta \Phi )}{|I^{+}{|}^{2}+|I^{-}{|}^{2}}\right).$$

where a narrow CPA peak can be obtained only when three conditions are satisfied: (1) |*r*| = |*t*|, (2) cos(Δ*φ*1)cos(Δ*Φ*) = −1, and (3) |*I*^+^| = |*I*^−^|.

To realize the sensing strategy of CPPC, polarization conversion from LPWs to CPWs is needed to produce PMPs (*AR* = 0 dB), where the specific formula of *AR* is expressed as follows [31]:

(18) $$\mathrm{AR}={\left(\frac{|Q_{\mathrm{TE}}{|}^{2}+|Q_{\mathrm{TM}}{|}^{2}+\sqrt{a}}{|Q_{\mathrm{TE}}{|}^{2}+|Q_{\mathrm{TM}}{|}^{2}-\sqrt{a}}\right)}^{\frac{1}{2}}.$$

where

(19) $$a=|Q_{\mathrm{TE}}{|}^{4}+|Q_{\mathrm{TM}}{|}^{4}+2{|Q}_{\mathrm{TE}}{|}^{2}{|Q}_{\mathrm{TM}}{|}^{2}\cos(2\Delta \varphi ),$$

(20) $$Q_{\mathrm{TE}(\mathrm{TM})}^{\pm }=|Q^{\pm }{|}^{2}.$$

The CPPC sensing strategy is innovative, which is to observe the frequency shift in PMPs to detect the changes in RI of the detected object. It is important to produce enough and proper PMPs for the sensing strategy of CPPC, meaning the scattering EM waves must meet the requirements of certain polarization states. When the EM waves are LPWs, the Δ*φ* = 180° ± 180°*n* (*n* = 0, 1, 2…) [20]. If EM waves are CPWs, the Δ*φ* must satisfy 90° ± 180°*n* (*n* = 0, 1, 2…) and *AR* is less than 3 dB [20], which can be further divided into RHCPWs or left-handed circular polarization waves according to the Δ*φ* = +90° or −90°. If the EM waves do not meet the above situations, the polarization form of EM waves is elliptical polarization waves (EPWs) [20]. However, for the research of CPWs, more attention is paid to the value of *AR* (less than 3 dB) because the calculation of AR involves the Δ*φ* of the output EM waves, which can be referred to Equations (18)–(20). If the *AR* of EM waves is smaller than 3 dB, the Δ*φ* is usually near 90° ± 180°*n* (*n* = 0, 1, 2…), meaning the motion trajectory at the end of the electric field vector related to EM waves is approximately circular and the state of EM waves is CPWs. At this time, the Δ*φ* can be not strictly equal to 90° ± 180°*n* (*n* = 0, 1, 2…), but the *AR* must remain less than 3 dB. Further, according to Equations (18)–(20), the *AR* is equal to 0 dB only when the Δ*φ* is strictly 90° ± 180°*n* (*n* = 0, 1, 2…), where the output EM waves can be defined as standard CPWs with the motion trajectory at the end of the electric field vector being a standard circle. Therefore, through converting the incident LPWs to CPWs, enough and proper PMPs, used for the sensing function, can be obtained, representing the output EM waves are standard CPWs. Notably, the state of EM waves output from port 2 is researched in this paper.

## 3. Results and Discussion

Because the sensing application of CPPC and CPA is the key point in this paper, it is crucial to use the *Q*, *S*, figure of merit (*FOM*), and detection limit (*DL*) to evaluate the detection performance of the VP. The *f_T_* and *FWHM* are defined as the resonant frequency and half-height width of the resonant peak. Also, the Δ*f* and Δ*n* are on behalf of the changes in the frequency used for detection and the physical quantities of detected biomass, respectively. So, the formulas of *Q*, *S*, *FOM*, and *DL* can be written as follows [32,33]:

(21) $$Q=\frac{f_{T}}{\mathrm{FWHM}},$$

(22) $$S=\frac{\Delta f}{\Delta n},$$

(23) $$\mathrm{FOM}=\frac{S}{\mathrm{FWHM}},$$

(24) $$\mathrm{DL}=10\log\frac{f_{T}}{20\cdot S\cdot Q}.$$

Considering the lack of actual funds and the harshness of the experimental conditions for medium- and high-frequency terahertz, the work in this paper focuses on the theoretical research, where the specific experiment flow is added in Section S1 of the Supplemental Material. Also, for the manufacturing of the VP, the wet anisotropic etching method can be used, the special description of which is in Section S2 of the Supplemental Material.

**Figure 2:**
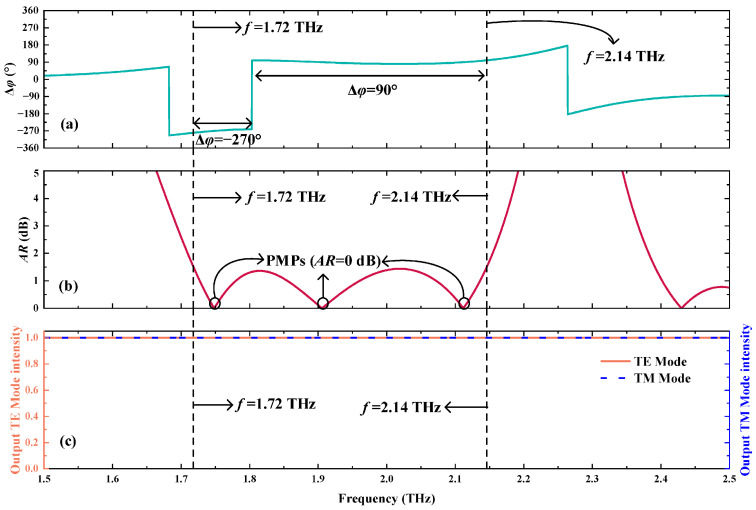
The output EM waves are RHCPWs when the *T_e_* = 300 K and *B* = 0.98 T. (**a**) Δ*φ.* (**b**) *AR*. (**c**) The output wave intensity under the TE and TM modes.

Based on the transfer matrix method of the layered structure theory [20], the propagation characteristics of EM waves can be calculated, with specific contents detailed in Section 2. When the *T_e_* is fixed as 300 K and *B* is equal to 0.98 T, a certain polarization conversion from LPWs to RHCPWs can be achieved in the range of 1.72 THz~2.14 THz. As shown in Figure 2a,b, the Δ*φ* is 90° or −270° and *AR* is smaller than 3 dB within the frequency coverage of 1.72 THz~2.14 THz, representing the polarization state of the output EMs is RHCPWs. Notably, in Figure 2b, there are three frequency points of *AR* being 0 dB, called PMPs, in the operating frequency band, which are regarded as the manifestation of CPPC [6]. Here, the state of the output EM waves is standard CPWs under the case of meeting the conditions of PMPs. According to Equations (18), (19) and (20), to make the *AR* of EM waves equal to 0 dB, the Δ*φ* must remain 90° ± 180°*n* (*n* = 0, 1, 2…), and the amplitude of electric fields related to TE waves is the same as that of TM waves. So, if the output EM waves do not satisfy the conditions of PMPs, the state of the output EM waves can be CPWs, LPWs, or EPWs, the *AR* of which can not form an obvious and easy-to-distinguish frequency peak. However, owing to the fact that biosensing, based on EM waves, usually needs to establish a linear relationship between the displacement of a detecting frequency peak and changes in the detected biomass, there are only the PMPs suitable for biosensing based on the CPPC strategy. The specific discussion of PMP used for detection will be illustrated below. Moreover, as shown in Figure 2c, when the output EM waves are RHCPWs, the energy loss of EMs under the TE and TM modes is nearly approaching 0, allowing polarization conversion from LPWs to RHCPWs to have an extremely high polarization conversion rate based on the interference of coherent EM waves.

**Figure 3:**
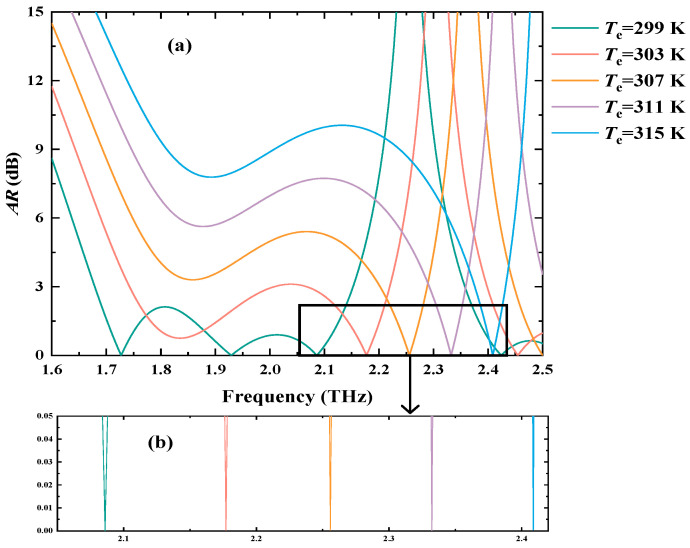
The diagrams of the PMP being applied for the detection of *T_e_*. (**a**) The shift changes in PMP with *T_e_* varying from 299 K to 315 K. (**b**) The enlarged image of the variations in PMP.

The core of realizing the detection application of PMP is to form a bijective relationship between the shift in PMP and external environment parameters [6], where the changes in environment parameters can produce the variations in the local phase related to RHCPWs to further make the frequency shifts of PMP. Considering the temperature tunability of InSb, temperature can serve as an important factor to adjust the polarization states of the output EM waves, allowing the proposed VP to have great potential in realizing temperature detection by the frequency shift in PMP. When *n*_E_ is fixed as 1.35 and *B* is 0.98 T, Figure 3a displays the specific connection between the *T_e_* and *AR* of output EM waves. With *T_e_* altering from 299 K to 315 K, the frequency of the PMP will linearly shift from 2.08597 THz to 2.40874 THz. The enlarged image of PMP is demonstrated in Figure 3b, where the *AR* of PMP is almost 0 dB, and the shift in the frequency related to PMP builds a linear relationship with the variations in *T_e_*. Therefore, it is feasible for the designed VP to fulfill the temperature detection by observing the frequency shift in PMP.

**Figure 4:**
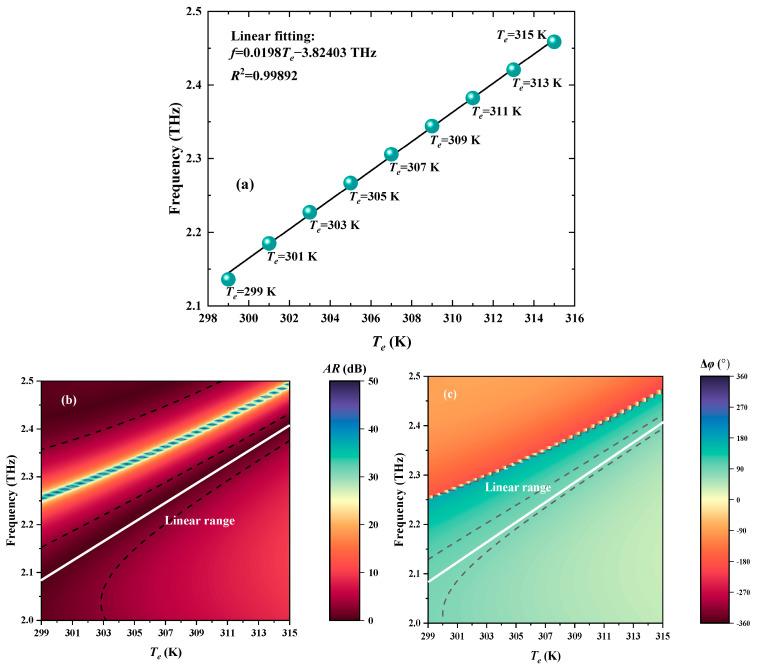
The *T_e_* sensing performance of the proposed VP. (**a**) The linear fitting curve for *T_e_* and PMP. (**b**) The influences of *T_e_* on *AR* of output EM waves. (**c**) The effects of Te on Δ*φ* of output EM waves.

To illustrate the possibility of realizing the *T_e_* detection by the PMP method, the *T_e_* and frequency of PMP can be linearly fitted, which is indicated in Figure 4a. The linear fitting relationship (LPR) can be described as *f* = 0.0198*T_e_* − 3.82403 THz with the *S* being 0.0198 THz/K. Moreover, the determination coefficient *R*^2^ is as high as 0.99892 [34], where the quality of the linear fitting is better when the *R*^2^ is closer to 1 [34]. Therefore, the VP can be effectively employed for the accurate measurement of temperature with high sensing performance. In addition, the white lines, representing linear range, in Figure 4b,c obviously show the potential of utilizing PMP for temperature detection, which has high sensitivity and a wide detection range. Under the frequency of PMP, the standard RHCPWs are continuously output by the proposed VP, proving the effectiveness of the detection based on CPPC.

Since cancerous cells usually have more protein contents than their corresponding normal cells, there is a RI difference between a cancerous cell and a normal cell [8], which can act as the basis for the detection of early cancerous cells. Table 1 concludes and shows six different types of cells, providing specific data about RI when a certain cell is normal or cancerous. It is particularly emphasized that the chosen cancerous cell liquid is 80% concentration and the chosen normal cell liquid is 30–70% concentration [35], which are to be injected into the detected layers E.

**Figure 5:**
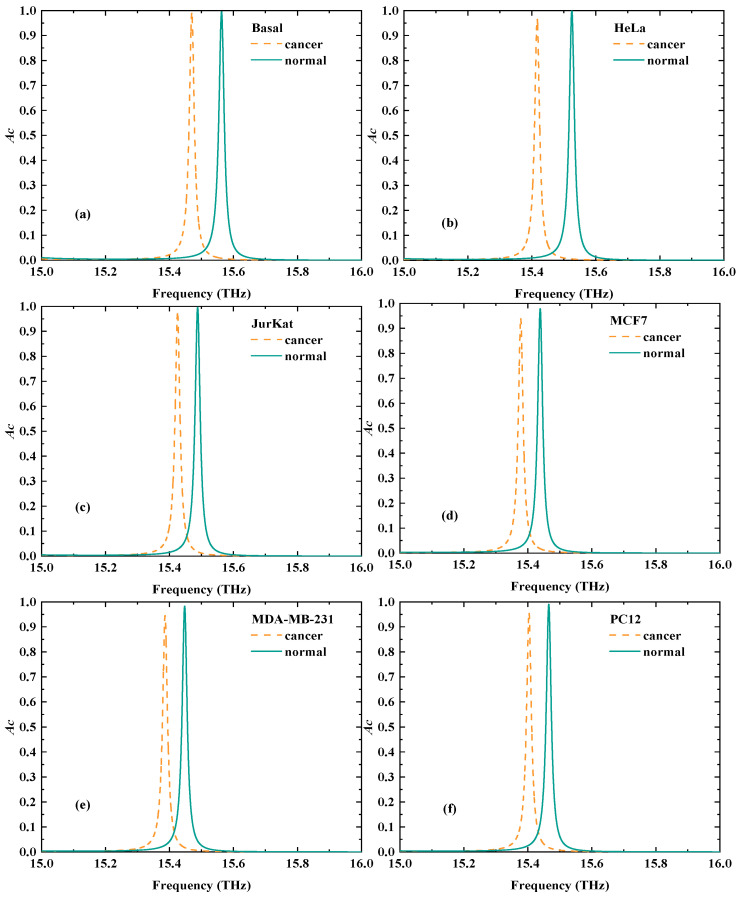
The comparison charts of cancerous and normal cells. (**a**) Basal. (**b**) HeLa. (**c**) JurKat. (**d**) MCF7. (**e**) MDA-MB-231. (**f**) PC12.

When the working state of the suggested VP is switched from CPPC to CPA, the *T_e_* is fixed as 300 K, and *B* at 0.5 T. At this time, an extremely narrow CPA peak can be generated by the VP within 15 THz–16 THz. Given that the condition of the zero amplitude of output fields must ensure that the eigenvalues of the scattering matrix related to CPA is zero when the amplitude of input fields is not zero [5], the CPA peak tends to be very sensitive to the variations in the external environment, making the detection of cancerous cells possible. The specific situations are demonstrated in Figure 5a–f, where the frequency of the CPA peak corresponding to normal cells is all larger than the frequency of the CPA peak related to cancerous cells.

**Figure 6:**
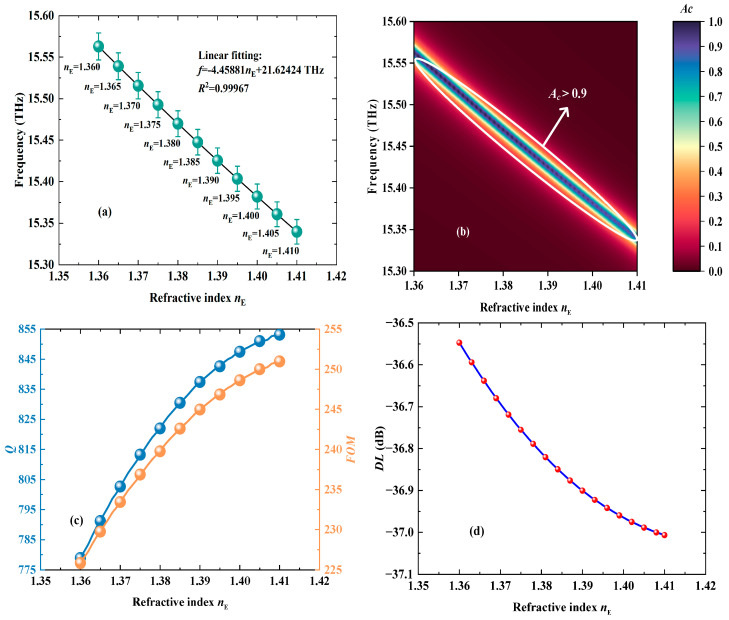
The diagrams of the detection performance of cancerous and normal cells. (**a**) The LFR between the RI and the frequency of the CPA peak. (**b**) The three-dimensional fitting diagram when the *n*_E_ changes from 1.36 RIU to 1.41 RIU. (**c**) The variations in *Q* and *FOM* with the *n*_E_ increasing from 1.36 RIU to 1.41 RIU. (**d**) The changes in *DL* as the *n*_E_ alters from 1.36 RIU to 1.41 RIU.

Through linearly fitting the relationship between the RIs of cancerous and normal cells and the frequency shift in the CPA peak, a LFR can be obtained and displayed in Figure 6a. Notably, Figure 6a contains error bars to show the *S* and linearity of the VP to the detection of cancerous cells, where the RIs of cancerous cells are assumed to fluctuate by 0.25% overall because the electromagnetic devices are sensitive to parameter changes in the VP. The LFR can be expressed as *f* = −4.45881*n*_E_ + 21.62424 THz, and the related *S* is determined as −4.45881 THz/RIU. The excellent linearity of detecting cancerous cells can be fully shown by the relevant *R*^2^ of 0.99967. Also, in Figure 6b, the values of the CPA peak, employed for detection, all remain larger than 0.9, indicating the sensing stability of cancerous cells based on the proposed VP. To further evaluate the detection performance of the VP, the *Q*, figure of merit (*FOM*), and detection limit (*DL*) are introduced. The larger *Q* and *FOM* are on behalf of the better detection performance. The *DL* is the opposite of *Q* and *FOM*. As demonstrated in Figure 6c,d, the minimum *Q* and *FOM* are equal to 778.92 and 225.84, respectively, which both become larger with the RI increasing. Also, the *DL* will decrease when the RI increases, the maximum value of which is −36.54 dB. Therefore, the proposed VP has an excellent detection performance in distinguishing cancerous and normal cells.

Viruses are the main cause of many different diseases, such as influenza and HIV/AIDS, which can multiply millions of new viral particles in the host cells [36]. Therefore, it is necessary to detect the invasion of viruses as soon as possible for timely treatment. The proposed VP in this paper has the advantages of fast response, being user-friendly, and no pollution in the field of the virus detection. Here, COVID-19, known as the recent global pandemic, is chosen as the detected object of the VP to highlight the potential in the field of virus detection. According to the existing research [37], there are five different RIs corresponding to five different COVID-19 SCs in the phosphate-buffered solution, which are detailed in Table 2. Notably, the relationship between RI and COVID-19 SC in Table 2 is based on the test data of Ref. [37], where the test data are suitable for the detection environment in this paper. Moreover, according to Ref. [38], the RI of the COVID-19 solution will linearly change with the COVID-19 SC increasing, allowing the linear connection between RI and COVID-19 SC to be obtained through the linear fitting method. Then, with the specific RI of a COVID-19 solution being tested, the corresponding COVID-19 SC can be derived from the linear fitting relationship between RI and COVID-19 SC.

**Figure 7:**
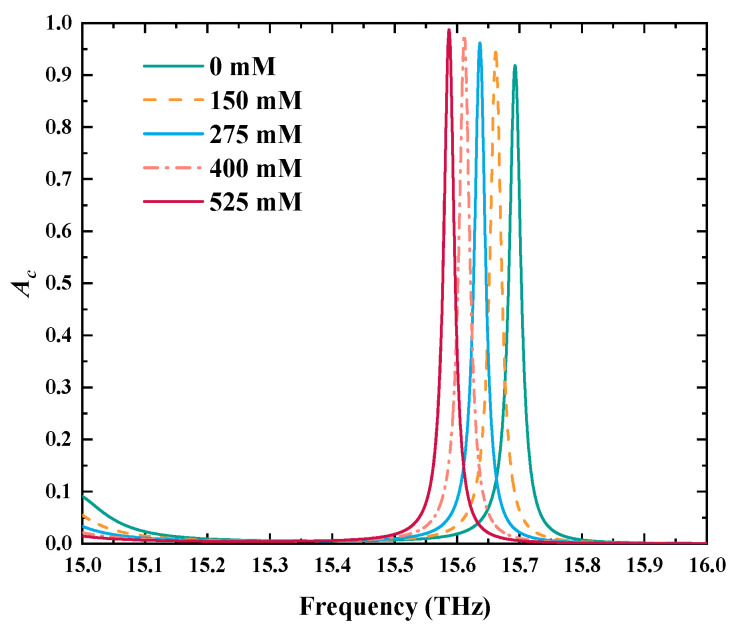
The changes in the CPA peak, used for detection, under five different COVID-19 SCs.

**Figure 8:**
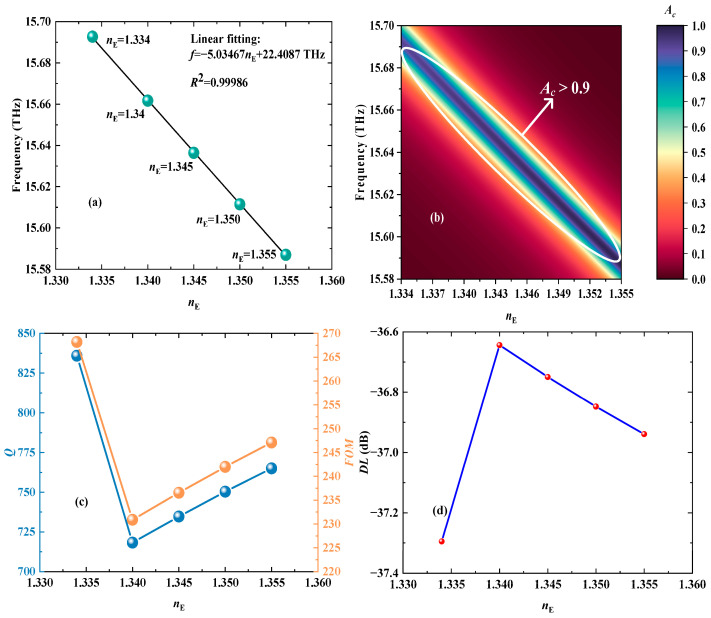
The drawings of the detection performance of five different RIs corresponding to COVID-19 SCs. (**a**) The LPR between the RI and the frequency of the CPA peak. (**b**) The three-dimensional fitting diagram when the *n*_E_ changes from 1.334 RIU to 1.355 RIU. (**c**) The variations in *Q* and *FOM* with the *n*_E_ increasing from 1.334 RIU to 1.355 RIU. (**d**) The changes in *DL* as the *n*_E_ alters from 1.334 RIU to 1.355 RIU.

The principle of detecting COVID-19 SC is to map the concentration changes to the RI variations. Therefore, the essence of detecting COVID-19 SC is the detection of changes in RIs of the detected layers. When the phosphate-buffered solution of COVID-19 is injected into the detected layers I, the *T*_e_ and *B* are 300 K and 0.5 T, respectively, where a narrow CPA peak is employed to sense the changes in COVID-19 SC. As revealed in Figure 7, as the COVID-19 SC increases from 0 mM to 525 mM, the related CPA peak will shift toward a lower frequency range. In addition, as shown in Figure 8a,b, the RIs of five different COVID-19 SCs are linearly fitted with the frequency of the CPA peak, the LFR of which can be determined as *f* = −5.03467*n*_E_ + 22.4087 THz with the *S* being −5.03467 THz/RIU. Moreover, the values of the CPA peaks are greater than 0.9 in the RI range of 1.334–1.355, and the relevant *R*^2^ is as high as 0.99986, meaning the VP has a good linearity and stability in detecting COVID-19 SCs. Moreover, as indicated in Figure 8c,d, the minimum *Q* and *FOM* are equal to 718.32 and 230.88 RIU^−1^, respectively. The maximum *DL* is −36.64 dB. Hence, the detection performance of COVID-19 SC is absolutely excellent, certifying the great potential of the designed VP in the field of detecting viruses.

To highlight the advantages of the proposed VP more obviously, Table 3 plainly concludes several studies about the sensing strategy in recent years, especially in the biosensing field. In Table 3, the “yes” stands for the existence of a certain nature, and the “no” is a reference to the inexistence of a certain feature or data in the relevant research. Moreover, all elements are placed in a fixed order. For instance, the detected physical quantities and research data correspond in sequence from top to bottom. For the improvement of research data, the same physical quantities are compared. If the detected physical quantities between other research and the proposed VP are different, the improvement of research data is calculated by the total average values of the VP. By comparing these studies with the designed VP, it is clear to find that the VP not only exhibits excellent detection performance of the temperature, cancerous cells, and COVID-19 SCs but also owns two unique detection strategies of CPA and CPPC with a multiscale sensing, which can provide an ideal method to realize multiscale and multi-biomass sensing and own great potential in the detection of the tiny changes in physical quantities, especially in the biosensing field.

## 4. Conclusions

To sum up, the designed VP is based on the interference of coherent waves to realize multiscale and multi-biomass sensing, with sensing strategies including CPPC and CPA. When the *T_e_* and *B* are fixed as 300 K and 0.98 T, polarization conversion from LPWs to RHCPWs can be obtained in the range of 1.72~2.14 THz, causing three PMPs. By observing the frequency shift in the chosen PMP with the *T_e_* changing, the detection of *T_e_* can be effectively realized in the detection coverage of 299 K~315 K. The related *S* is 0.0198 THz/K. When the *T_e_* is fixed as 300 K and *B* is altered to 0.5 T, the state of the VP is switched from CPPC to CPA. The sensing ranges of cancerous cells and COVID-19 SCs are 1.36 RIU~1.41 RIU and 0 mM~525 mM (1.334 RIU~1.355 RIU) with the *S* being −4.45881 THz/RIU and −5.03467 THz/RIU, respectively. The average *Q*, *FOM*, and *DL* of detecting cancerous cells are equal to 825.36, 241.11 RIU^−1^, and −36.83 dB. Also, the relevant *Q*, *FOM*, and *DL* of detecting COVID-19 SCs are 760.85, 244.94 RIU^−1^, and −36.89 dB. Therefore, with the function of multiscale and multi-biomass sensing, the proposed VP not only has great potential in the field of biosensing but also provides essential support for research in expanding the detection scale.

## Figures and Tables

**Table 1:** RIs of six different types of cells, including normal and cancerous cells [35].

Cell Type	Basal	HeLa	JurKat	MCF7	MDA-MB-231	PC12
Normal	1.36	1.368	1.376	1.387	1.385	1.381
Cancerous	1.38	1.392	1.39	1.401	1.399	1.395

**Table 2:** Five different RIs under five different COVID-19 SCs [37].

COVID-19 SC (mM)	RI
0	1.334
150	1.34
275	1.345
400	1.35
525	1.355

**Table 3:** The summaries of the past studies in the biosensing field, compared with the presented VP, where “yes” stands for the existence of a certain feature and “no” represents the inexistence of a certain characteristic or data in the relevant research.

Refs.	[15]	[16]	[18]	[19]	[39]	[40]	This Work
Detected quantities	Cancer	Cancer	Virus	Virus	RI	Isoquercitrin	Temperature
	Blood						Cancer
							COVID-19 SCs
Sensing range	1.36–1.399 RIU	1.35–1.39 RIU	1.334–1.344 RIU	1.373–2.049 RIU	1–1.6 RIU	1.333–1.385 RIU	299–315 K
	1.33–1.40 RIU		1.334–1.374 RIU				1.36–1.41 RIU
							0–525 mM
*Q*	7.11	61.75	no	430.52	6.55	28.64	no
	37.33		no	371.42			825.36
							760.85
Improvement of *Q* (%)	11,508.43%	1236.62%	no	76.73%	12,008.47%	266.92%	no
	2024.58%			104.84%			
*FOM*	0.2 RIU^−1^	7.5 RIU^−1^	0.377 RIU^−1^	480 RIU^−1^	no	20 RIU^−1^	no
	16.8 RIU^−1^		0.283 RIU^−1^	293.8 RIU^−1^			241.11 RIU^−1^
							244.94 RIU^−1^
Improvement of *FOM* (%)	120,455.00%	3114.8%	64,870.82%	−48.97%	no	1115.16%	no
	1346.58%		86,451.24%	−16.63%			
*DL*	no	no	no	no	no	−29.95 dB	no
							−36.83 dB
							−36.89 dB
Sensing strategies	Surface plasmon wave	Ring resonator	Polarization conversion rate	Resonant frequency	plasmon induced transparency	Resonance frequency	CPPC
							CPA
							CPA
Multiscale	no	no	no	no	no	no	yes

## Data Availability

Samples of the compounds are available from the authors.

## Supplementary Materials

The following supporting information can be downloaded at: https://www.mdpi.com/article/10.3390/bios15080516/s1, Figure S1: (a) The experiment flow of the VP. (b) The device for generating a spatial magnetic field; Figure S2: The fabrication flow of the proposed VP based on the wet anisotropic etching approach. References [41,42,43,44,45,46,47,48,49] are cited in the Supplementary Materials.
