# Supplementary material for: Theory Design of a Virtual Polarizer with Multiscale and Multi-Biomass Sensing
Source: Biosensors (Basel). 2025 Aug 8;15(8):516. doi: 10.3390/bios15080516 (PMC12384523; doi:10.3390/bios15080516)
Supplement: Supplementary file 1 [file biosensors-15-00516-s001.zip › biosensors-3788490-supplementary.pdf]

biosensors

Supplementary Material

Theory Design of a Virtual Polarizer with Multiscale and Multi-Biomass Sensing

Chuanqi Wu and Haifeng Zhang *

College of Electronic and Optical Engineering & College of Flexible Electronics (Future Technology), Nanjing University of Posts and Telecommunications, Nanjing 210023, China; b22021517@njupt.edu.cn

* Correspondence: hanlor@163.com or hanlor@njupt.edu.cn

S1. The experiment setup of the VP with multiscale and multi-biomass sensing

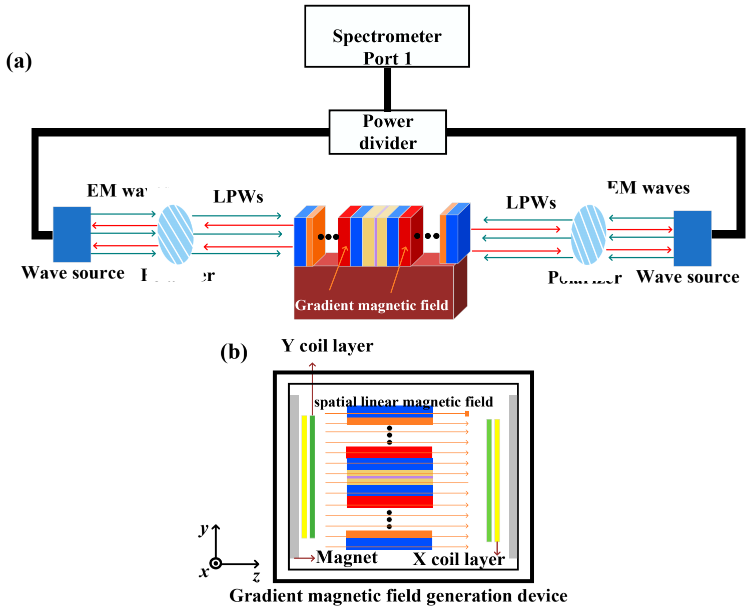

Figure S1. (a) The experiment flow of the VP. (b) The device for generating a spatial magnetic field.

Due to the fact that the principle of the proposed virtual polarizer (VP) is based on the interference of EM waves, the key point of the assumed experiment is to ensure the generation of coherent waves. Referring to the present Refs. [1-4], the total experiment must occur in the high-frequency THz anechoic chamber to prevent the interference from environmental factors, where the experiment flow is detailed in Figure S1(a). To ensure the generation of coherent waves, two identical wave sources are symmetrically placed on both sides of the designed VP. Notably, the chosen sources are not only employed to produce coherent waves but also used to receive the EM waves processed by the VP. Then, the EM waves received by the wave sources will be transferred to a power divider and port 1 of a spectrometer through identical coaxial cables. Moreover, the polarizers are used to ensure the EM waves incident on the VP are LPWs. Finally, by analysing the results in the spectrometer, the related functions of the VP can be proved. Due to the mag-

 https://doi.org/10.3390/bios15080516

netic field tunability of InSb, the realization of a magnetic field is also important. The gradient magnetic field method is taken to modulate the VP, which has been studied theoretically and applied in reality [5, 6]. As exhibited in Figure 10(b), the principle is to magnetize the magnetic material, placed in a loop, by the magnetic field generated from the X and Y coil layers with a current. Then, a spatial linear magnetic field, along the *z*-axis, can be employed to modulate the VP, satisfying the needs of the directional magnetic field.

S2. The manufacturing process of the designed VP

With the multiscale and multi-biomass sensing, the designed virtual polarizer (VP) is a layered structure, which can be manufactured through the wet anisotropic etching method [7]. At present, the wet anisotropic etching method has been widely employed for the fabrication of certain structures [8, 9]. Given that there is no influence of the width and length of the VP on the propagation of EM waves, the thicknesses of each layer in the VP are the key points in the fabrication process. Moreover, referring to Ref. [7-9], the wet anisotropic etching method has been successfully utilized for the manufacturing of an array of silicon nanowires with an aspect ratio of 93. The corresponding height and diameter are 51 μm and 550 nm, proving that the wet anisotropic etching method is suitable for the fabrication of the VP. The related materials include the photoresist, silicon dioxide (SiO_2), (110) silica wafer, buffered hydrofluoric acid (BHF), and aqueous solution of 44% potassium hydroxide (KOH), where the specific manufacturing process of the VP is exhibited in Figure S1.

Step (a): The (110) silica wafer serves as the fabrication substrate, and the SiO_2 is positioned on the upper side of the silica wafer. Finally, the photoresist is applied on the surface of the SiO_2, benefiting the laser lithography.

Step (b): After the laser lithography, the initial vertical grooves will be produced in the photoresist, with the purpose of determining the insertion positions of different dielectrics marked by different colors.

Step (c): The pattern related to the initial vertical grooves can be transferred into the SiO_2 through the etching of BHF for the SiO_2.

Step (d): After finishing the transfer of the patterns, the residual photoresist needs to be completely cleared to prevent the influence of impurities on the SiO_2 and Si.

Step (e): At 85 ℃, the aqueous solution of 44% KOH is utilized to etch the Si wafer to make the patterns of the vertical grooves transferred in the Si wafer, where the thermally grown SiO_2 layers will act as hard masks.

Step (f): Finally, different dielectric layers are inserted into the correct positions in the silica wafer. Notably, the silica wafer can be considered an excellent structure with its length and width being extended freely, whose characteristics can be consistent with those from theoretical research [7-9].

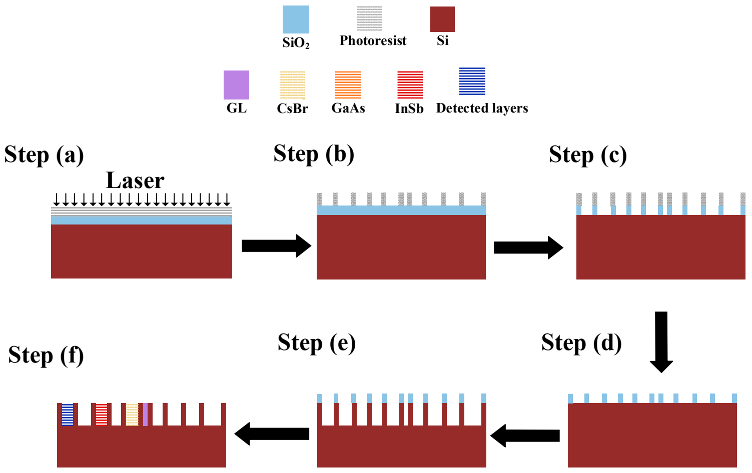

Figure S2. The fabrication flow of the proposed VP based on the wet anisotropic etching approach.

References

1. Li, S.; Luo, J.; Anwar, S.; Li, S.; Lu, W.; Hang, Z. H.; Wang, C. Broadband perfect absorption of ultrathin conductive films with coherent illumination: Superabsorption of microwave radiation. *Physical Review B* **2015** 91, 220301.
2. Zhang, X.; Meng, C.; Yang, Z. Wave manipulations by coherent perfect channeling. *Scientific Reports* **2017** 7, 13907.
3. Schmidt, C.; Sudzius, M.; Meister, S.; Fröb, H.; Leo, K. Controllable coherent absorption of counterpropagating laser beams in organic microcavities. *Applied Physics Letters* **2020** 117, 053301.
4. Urade, Y.; Nakata, Y.; Nakanishi, T.; Kitano, M. Coherent absorption of terahertz pulses by a checkerboard metasurface. *In 2015 40th International Conference on Infrared, Millimeter, and Terahertz waves (IRMMW-THz) (pp. 1-2). IEEE* **2015**.
5. Xuan, L.; Kong, X.; Wu, J.; He, Y.; Xu, Z. A smoothly-connected crescent transverse gradient coil design for 50mT MRI system. *Applied Magnetic Resonance* **2021** 52, 649-660.
6. Ma, Y.; Zhang, H. Wide-angle energy steering and magnetic information detection-coding of stacked ferrite-based elements in the gradient magnetic domain. *Optics & Laser Technology* **2022** 156, 108544.
7. Guo, S.; Hu, C.; Zhang, H. Ultra-wide unidirectional infrared absorber based on 1D gyromagnetic photonic crystals concatenated with general Fibonacci quasi-periodic structure in transverse magnetization. *Journal of Optics* **2020** 22, 105101.
8. David, C.; Bruder, J.; Rohbeck, T.; Grünzweig, C.; Kottler, C.; Diaz, A.; Pfeiffer, F. Fabrication of diffraction gratings for hard X-ray phase contrast imaging. *Microelectronic Engineering* **2007** 84, 1172-1177.
9. Tolmachev, V. A.; Granitsyna, L. S.; Vlasova, E. N.; Volchek, B. Z.; Nashchekin, A. V.; Remenyuk, A. D.; Astrova, E. V. One-dimensional photonic crystal obtained by vertical anisotropic etching of silicon. *Semiconductors* **2002** 36, 932-935.

Disclaimer/Publisher's Note: The statements, opinions and data contained in all publications are solely those of the individual author(s) and contributor(s) and not of MDPI and/or the editor(s). MDPI and/or the editor(s) disclaim responsibility for any injury to people or property resulting from any ideas, methods, instructions or products referred to in the content.
